# Quality of life outcomes from a randomized controlled trial of patient navigation in Latina breast cancer survivors

**DOI:** 10.1002/cam4.3272

**Published:** 2020-09-26

**Authors:** Amelie G. Ramirez, Edgar Muñoz, Dorothy Long Parma, Arely Perez, Alfredo Santillan

**Affiliations:** ^1^ Department of Population Health Sciences UT Health San Antonio San Antonio TX USA; ^2^ The Mays Cancer Center UT Health San Antonio MD Anderson Cancer Center San Antonio TX USA

**Keywords:** behavioral science, breast cancer, clinical trials, quality of life

## Abstract

**Introduction:**

Breast cancer survivorship is a life‐long process involving challenges to health‐care communities and individuals, especially Latinas. Patient Navigation has shown some success in meeting these challenges. The purpose of this study was to compare the effects of an enhanced Patient Navigation program (Intervention; PN+) vs Control (PN) over time on general cancer and breast cancer‐specific quality of life (QoL) in Latina breast cancer survivors (BCS).

**Methods:**

We conducted a 2‐year, two‐arm randomized controlled trial of the “Staying Healthy” program among Latina BCS. The design compared PN+ vs PN over time. We recruited 60 patients into each study arm and randomized them by sequential numerical assignment. PN+ participants received culturally tailored educational materials and active, personalized Patient Navigation services, including phone calls, transportation, and care coordination. PN participants were navigated only upon request. Primary outcomes included general cancer (Functional Assessments of Cancer Therapy [FACTS]‐G) and breast cancer‐specific (FACT‐B) QoL.

**Results:**

PN+ participants had significantly improved QoL measures compared to PN at 6‐month follow‐up on all subscales (*P*‐values .007‐.04) except physical well‐being (PWB; *P* = .11). Intervention effect size coefficient (standard error) for FACT‐G overall was 7.9 (3.1); *P* = .01. For FACT‐B, it was 10.9 (3.9); *P* = .006. Again, all subscales showed significant effects [range 1.7‐3.1 (0.8‐1.2); *P*‐values .006‐.04], except for PWB [1.5 (1.0); *P* = .16] and social/family well‐being (SWB) [2.1 (1.1); *P* = .06]. There were no differences between groups at baseline.

**Discussion:**

Multiple cultural, psychosocial, and socioeconomic variables contributing to these intervention effects will be addressed in future studies. As the national BCS population continues to increase, more Patient Navigation‐focused partnerships among patients, health‐care professionals, research groups, and community organizations are needed to improve BCS experiences. The Staying Healthy program has the potential to serve as a national survivorship care model for improving Latina BCS QoL.

## Graphical abstract

We evaluated the effect of a two‐year, two‐arm randomized controlled trial (the “Staying Healthy” program) on quality of life (QoL) among Latina breast cancer survivors (BCS). The design compared enhanced Patient Navigation (Intervention; PN+) versus usual (Control; PN) over time. PN+ participants had significantly improved quality of life measures compared to PN at 6‐month follow‐up.

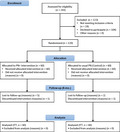

## 1 INTRODUCTION

Of the almost 17 million cancer survivors in the US in 2019, over 3.8 million were breast cancer survivors (BCS). Over 324 500 new cases, including ductal carcinoma in situ (DCIS), will be diagnosed in 2019‐2020.^1^, ^2^ Latina BCS have higher rates of late‐stage diagnosis relative to non‐Latina whites; these likely reflect disparities in both access to care and timely, high‐quality treatment.^3^, ^4^ The development of chronic conditions and disabilities, cognitive decline, and difficulty performing activities of daily living are part of the BCS survivorship experience and can lead to poorer quality of life (QoL).^5^, ^6^, ^7^, ^8^, ^9^ This is exacerbated in Latina populations, who have decreased knowledge of their disease and satisfaction with information provided,^10^ and experience psychosocial, cultural, and socioeconomic barriers,^11^ increased worry, and fear about cancer recurrence or metastasis,^12^, ^13^, ^14^ and other unmet psychological supportive care needs like uncertainty about the future.^14^

The complicated BCS experience underscores the need to intervene on, and accurately measure, QoL among BCS to identify ways to improve their cancer journeys. Although multiple general QoL instruments are available like the Short Form (SF‐36) and its derivatives (eg, SF‐12),^15^ the Functional Assessments of Cancer Therapy (FACTS) scales developed by Cella and colleagues have been routinely used to measure cancer survivors’ QoL for almost three decades.^16^ The 27‐item, self‐report FACT‐General (FACT‐G) is one of the most widely used instruments to assess overall adjustment to cancer treatment and survivorship.^17^ The FACT‐G assesses QoL along a Likert scale in four domains of well‐being: physical, social/family, emotional, and functional. The scale has been validated for use in multiple populations, including older cancer patients^18^, ^19^, ^20^ and early stage BCS.^21^ The FACT‐B, a version specific for BCS,^22^ adds questions to FACT‐G which address QoL issues that are common sequelae of breast cancer.^23^ FACT‐B has been used to measure factors associated with QoL in multiple ethnic groups, including Latina BCS.^8^, ^13^, ^24^

As an intervention to improve survivorship care, Patient Navigation has been a required part of standard care for all accredited Cancer Centers since 2016.^25^ This development was in recognition of its potential to assist survivors and their health‐care professionals (HCPs) in negotiating the complexities of the health‐care system to improve HCP recommendation adherence.^26^ Patient Navigators (hereafter referred to as *promotoras*) have included a multidisciplinary team of community health workers (CHWs) and trained professionals oriented to patient advocacy, such as oncology nurses.^27^, ^28^, ^29^ Nurses particularly excel at navigating patients through survivorship clinical care due to their knowledge of specific health systems, while CHWs often come from the same communities served by the Patient Navigation program, and thus share cultural features (eg, language and beliefs) with patients. Several trials have assessed the use of Patient Navigation in Latina BCS; it has successfully been used to reduce time to diagnosis and treatment, and increase informed decision‐making regarding clinical trial participation.^29^, ^30^, ^31^ However, studies have had conflicting results, and several studies have been hindered by methodological shortcomings like lack of robust evaluation strategies, control groups, adequate descriptions of *promotoras*, long‐term follow‐up assessments, or large, diverse samples.^32^, ^33^ In addition, few studies have focused on patient QoL or satisfaction with care.^34^, ^35^, ^36^ Prior work by Moreno and colleagues has shown direct and indirect relationships between satisfaction with care and QoL in a path model that involves aspects of patient self‐efficacy (managing patient‐HCP communication, psychological distress, social support, and social/recreational activities).^34^ Likewise, Ramirez and colleagues proposed a conceptual model wherein a specific type of enhanced Patient Navigation would improve QoL and treatment adherence among cancer survivors by decreasing unmet needs and increasing effective patient‐HCP communication and positive health behaviors.^36^ We adapted this model for our local BCS population and smaller study size. We also modified the Patient Navigation approach to one found successful in previous small studies.^29^, ^37^, ^38^, ^39^

The purpose of this study was to compare the effects of an enhanced Patient Navigation program (Intervention; PN+) vs Control (PN) over time on general cancer and breast cancer‐specific QoL in Latina BCS. We hypothesized that participants receiving PN+ would have significantly higher QoL, both general and breast cancer‐specific, after 6 months than those receiving PN. Our study is unique in that it is a randomized controlled trial focusing on Latina BCS and comparing two types of Patient Navigation as our Control and Intervention, compared to previous studies (pre‐2016) which compared it to usual care (ie, no navigation services). We believe that, considering the rising Latino demographic in the United States (predicted to comprise 31% of all Americans by 2060),^40^ such a focus constitutes a significant contribution to the Patient Navigation literature.

## 2 METHODS

### 2.1 Study design

We conducted a 2‐year, two‐arm randomized controlled trial of the “Staying Healthy” program among Latina BCS in San Antonio, Texas. The design compared enhanced Patient Navigation (PN+) vs usual PN (between‐groups) over time (within‐groups).

### 2.2 Study setting

Based on the US Census Bureau projections for 2016, the total population of the service area of San Antonio, Texas was approximately 1.439 million. The greater Bexar County population was 63.6% Latino.^41^ In 2016, ~19.5% of the county population lived below the poverty line.^42^ Poverty, which is associated with low income, low education, and lack of health insurance, is a critical factor affecting health. High poverty levels are associated with a lower proportion of cancers diagnosed at earlier stages, when more treatment options are available and survival rates are higher.^43^, ^44^

San Antonio also is home to a National Cancer Institute‐designated Cancer Center. South Texas's only academic cancer research and treatment facility, it serves 4.9 million people (~70% Latino; ~35% living below 150% of the federal poverty line)^42^, ^45^ in the high‐growth corridor of Central and South Texas that includes Austin, San Antonio, Laredo, and the Lower Rio Grande Valley. Our institution has established collaborations with oncology centers and community‐based organizations providing services to breast, colorectal, and prostate cancer survivors. We have existing community connections and a Patient Navigation program available in English and Spanish.

### 2.3 Participant eligibility and recruitment procedures

#### 2.3.1 Inclusion and exclusion criteria

Eligible participants had to be 18 or older, self‐identify as a Latina female, and have a primary (ie, first‐time) diagnosis of breast cancer, including stages I‐III and DCIS. Patients must have had no evidence of metastatic disease. Participants had to have completed their primary treatment (surgery, chemotherapy, radiation, or a combination; or hormonal therapy) between 3 and 36 months prior to study recruitment and must not be on any ongoing neoadjuvant therapy (chemotherapy or radiation). They must also have had a deficit in either cancer screening (in need of a pap smear or colonoscopy according to the US Preventive Services guidelines) or a positive comorbidity screening (BMI of ≥25, diagnosis of diabetes or a random glucose level of ≥200 mg/dL, a diastolic blood pressure ≥90 mmHg, or a systolic reading ≥140 mmHg, or is a current smoker). Patients must also have shown evidence of verbal fluency in either English or Spanish and had to be available for follow‐up during the 6‐month intervention period.

#### 2.3.2 Recruitment

This study was approved by the University's Institutional Review Board (IRB) and conforms to the US Federal Policy for the Protection of Human Subjects. We recruited all patients from the Cancer Center's Breast Clinic. Our collaborating physicians and their staff screened potential participants. The research nurse first scanned the Breast Clinic's electronic medical record to identify patients meeting basic eligibility criteria. Potential participants identified by the nurse were then contacted at their clinic visit or by telephone to conduct a formal eligibility screening. A University IRB‐approved informed consent was administered once a patient was eligible and verbally agreed to participate. Participants received two $25 gift cards to a local supermarket if they completed baseline and post assessments and were available over the course of the 6‐month follow‐up period.

#### 2.3.3 Patient navigators

We recruited three bilingual Patient Navigators (*promotoras)* for this study. One was a CHW certified and recruited through the local CHW Association. The other two were nurses working at the Cancer Center in research and clinical positions. We determined Spanish fluency by credentials (ie, Associate Degree in Spanish, former employment as a bilingual elementary school teacher) and second‐generation Mexican American status with Spanish‐speaking‐only parents.

*Promotoras* were trained in Motivational Interviewing, a behavior‐changing approach first developed in the counseling and addiction community, and more recently applied in the health care field in the form of brief behavioral counseling.^46^, ^47^ Training and consultation were provided by a certified member of the Motivational Interviewing Network of Trainers.^48^ *Promotoras* also underwent training to increase their knowledge base of all resources to be used in the intervention, as well as the processes involved in helping patients navigate the local health system, including appointments, referrals, etc. A list of all resources *promotoras* were trained to offer is shown in Table 1.

**TABLE 1:** Staying healthy resources

Resource type	Source organization	Title	Language
Cancer screening	American Cancer Society	*What Women Should Know About Cervical Cancer and the Human Papilloma Virus* ^a^	Spanish version available
		*Colorectal Cancer informational booklet*+ *DVD; tear‐off bookmark with MD‐directed questions* ^a^	
		*Cancer Facts for Women*	
	National Cancer Institute	*What You Need to Know About Cervical Cancer*	
		*What You Need to know about Cancer of the Colon and Rectum*	
Cancer survivorship	Livestrong Foundation	*The Road to Survivorship: Living After Cancer Treatment*	Spanish version available
Comorbidity screening	American Diabetes Association	*Are you At Risk For Type 2 Diabetes?* ^a^	Bilingual
		*Diabetes Advisor: Understanding Type 2 Diabetes* ^a^	Spanish version available
	American Heart Association	*Healthy Heart, Fast Guide*	
		*Managing Blood Pressure*	
	National Heart, Lung and Blood Institute	*Keep the Beat: Control Your High Blood Pressure (Healthy Hearts, Happy Homes)*	Bilingual
		*Do you know Your Cholesterol Levels? (Healthy Hearts, Happy Homes)*	
Community resources	University Cancer Center	*Maximizing Cancer Survivorship Evidence‐Based Exercise Program* ^a^	English only
		*Wellness Center Program Schedule (includes Zumba classes)* ^a^	
	Thrivewell Cancer Foundation	*Deriving Inspiration and Vitality through Activity (DIVA) Program* ^a^	
	Texas Diabetes Institute	*¡Salsa Caliente! group fitness class*	Spanish version available
	Patient Advocate Foundation	*Breast Cancer Resource Directory; Insurance/other resources*	English only
	BRACAnalysis	*Hereditary Breast & Ovarian Cancer: Beyond Risk to Options*	
Referrals	University healthcare providers	*colorectal and cervical cancer screening* ^b^	N/A
	San Antonio Cancer Center	*Wellness Center Program; Fitness Center*	
	Texas Diabetes Institute	*Salsa Caliente group fitness class, nutrition resources*	

^a^ Provided to all PN+ participants.

^b^ Offered to all PN+ participants identified from electronic medical record as requiring guideline‐recommended screening.

### 2.4 Study groups

We recruited 60 patients into each study arm. Control (PN) participants received a fact sheet of study services with *promotora* contact information and contact with a bilingual *promotora* during assessments. Additional navigation services were provided only upon participant request. In addition to PN services, intervention (PN+) participants received culturally tailored educational materials. Most materials were either available in both English and Spanish versions or were bilingual and were provided by national and local organizations. Local organizations were more likely to provide culturally relevant, bilingual and/or Spanish language options for participants, such as group salsa fitness classes (Table 1).

*Promotoras* also provided PN+ participants with regular personalized assistance including phone calls, home visits, transportation assistance, and coordination of targeted care. They educated participants on the importance of cancer screening, and scheduled appointments for screenings, exercise classes, and diabetes education and nutrition classes. They provided bus passes and/or accompanied participants to clinic appointments, provided translation services, and facilitated referral to community resources including the San Antonio Food Bank, educational opportunities at Alamo Community Colleges, and help with insurance applications for Medicaid, Medicare, and the Affordable Care Act.

**FIGURE 1:**
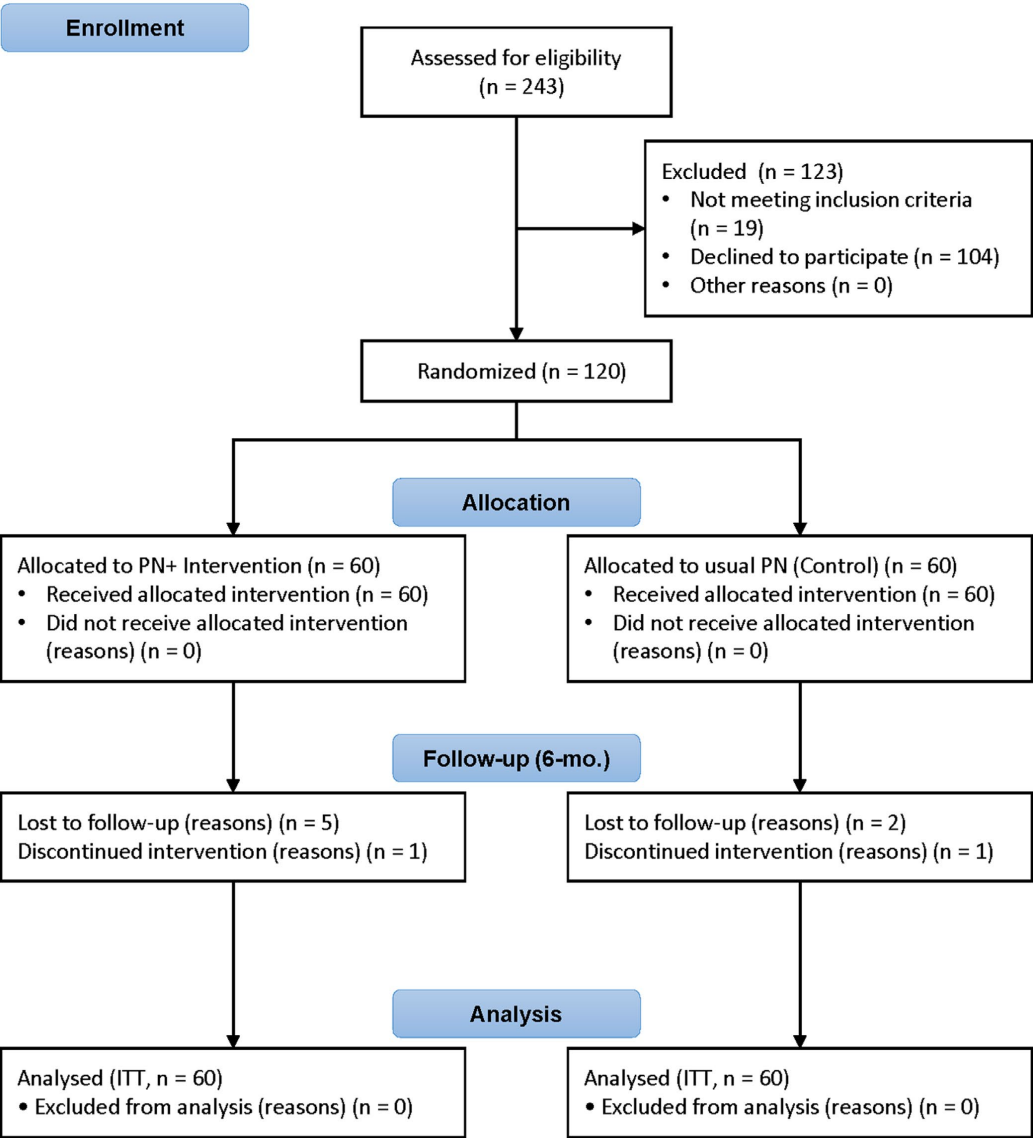
CONSORT flow diagram for the Komen “Staying Healthy” project. CONSORT, Consolidated Standards of Reporting Trials; ITT, intention to treat; PN, patient navigation

The intervention followed a PN model that has been described in more detail elsewhere.^49^ Figure 1 shows the Consolidated Standards of Reporting Trials (CONSORT) flow diagram.^50^, ^51^

### 2.5 Measures

*Promotoras* administered all assessments in‐person to all participants at the Breast Clinic, in space made available for the study. These assessments included a cancer‐specific QoL questionnaire, with scales addressing general cancer and site‐specific (breast) QoL. The survey was repeated at 6 months to see if PN services were beneficial in helping patients improve their QoL.

#### 2.5.1 Primary outcome measures (general cancer and breast cancer‐specific QoL)

We administered the FACT‐G to evaluate the general domains of QoL.^17^, ^19^ *Promotoras* asked participants to indicate the extent to which they agreed with statements such as "I have pain," "I feel ill," "I get emotional support from my family," "I get support from my friends," "I feel sad," "I feel nervous," "I am sleeping well," and "I am content with the quality of my life right now." We calculated a composite score for general QoL as well as subscale scores for specific QoL domains.

We also administered the FACT‐Breast (FACT‐B), which addresses QoL issues commonly experienced specifically by BCS.^22^, ^23^

### 2.6 Plan of analysis

#### 2.6.1 Sample size/power analysis

We estimated sample size based on a conservative hypothetical power calculation (0.90) for 100 participants (50 per group) in two equal groups measured at two time points (baseline and 6 months post), to detect a significant group × time modest effect size difference (*f* = 0.164, *α* = 0.05). We selected this power level to increase the probability of rejecting the null hypothesis and therefore reducing optimism bias. Our effect sizes were calculated from earlier studies on a mixed population of cancer survivors including BCS.^36^, ^49^ Our final baseline sample size per group was 120 (60 per group), considering an attrition rate of ~17%. All power calculations were done using G*Power version 3 for Windows.^52^

#### 2.6.2 Randomization

We randomized all participants, 1:1, either into the intervention (PN+) group or into usual care (PN) group. We preassigned randomization by identification and group number prior to the start of study recruitment. We generated the randomized schedule for 120 participants using SAS PROC PLAN, which was then loaded into the Research Electronic Data Capture system hosted at our institution.^53^ We gave participants numerical assignments as enrollment occurred. Randomization occurred at the end of the baseline assessment. This process aimed to create groups with equal characteristics to each other in respect to both socioeconomic and demographic variables and disease and medical variables. Both groups had access to a *promotora*, but the PN group had to request assistance for any navigation services they needed.

#### 2.6.3 Data analyses

We calculated means and standard deviations for all continuous variables and frequencies and percentages for categorical ones, overall and by experimental group. We used Fisher's exact test to compare categorical variables between PN+ and PN groups, and Mann‐Whitney test to compare continuous variables. The primary outcomes included changes in the FACT‐G score and the breast cancer‐specific score (FACT‐B) from the baseline at 6 months follow‐up. We calculated Cronbach's α reliability coefficient overall and by participant's preferred language for each outcome; it was 0.76‐0.9 for participants reporting English, and 0.74‐0.86 for those reporting Spanish as their primary language. We performed linear regression on all outcomes, regressing at 6 months on baseline scores and a binary intervention variable (1 = PN+ and 0 = PN). We used the coefficient (and its standard error) corresponding with the intervention term in this linear model as the estimate of the intervention effect for each outcome. We conducted sensitivity analyses on these estimates by adding demographic variables (ie, age, preferred language, and insurance) and intervention process indicators (ie, number of phone calls with a *promotora* and number of in‐person accompanied clinic/hospital visits) to the base linear model described above. The effect of missing values on the analysis was negligible; 7 of 120 participants were lost to follow‐up at 6 months, below our estimated attrition rate of 17%. We therefore did not conduct any type of imputation on the data. We conducted all analyses in Stata® version 15 (StataCorp)^54^ and SAS/STAT version 9.3 (SAS Institute, Inc). We used a significance level of .05 in all analyses.

## 3 RESULTS

Baseline demographic characteristics are shown in Table 2. Mean age of participants was 58.2 in the PN group and 56.4 in the PN+ group. Over 85% identified Mexican as their Latino heritage, and over 55% were born in the US Over 38% identified themselves as first generation. Over 60% were educated primarily in the US Of those educated outside the US, over half received less than a 12th grade education. Most participants either spoke only Spanish (37.5%) or both English and Spanish (36.9%). Almost half of participants in both groups were married, and over 50% had children. Over 40% of participants were insured via Medicaid, followed by 30% by other means.

**TABLE 2:** Baseline demographic characteristics by study group

Characteristic	Study group		All subjects	*P*‐value
	PN	PN+		
Marital status n (%)				.31^a^
Single	6 (10)	10 (16.7)	16 (13.3)	
Married	27 (45)	29 (48.3)	56 (46.7)	
Dating	0 (0)	1 (1.7)	1 (0.8)	
Separated	2 (3.3)	5 (8.3)	7 (5.8)	
Divorced	17 (28.3)	11 (18.3)	28 (23.3)	
Widowed	8 (13.3)	4 (6.7)	12 (10)	
Total	60	60	120	
Do you have children? n (%)				.42^a^
Yes	50 (83.3)	54 (90)	104 (86.7)	
No	10 (16.7)	6 (10)	16 (13.3)	
Total	60	60	120	
Age (y) (Self‐reported)				.32^b^
N	60	60	120	
Mean (SD)	58.2 (9.4)	56.4 (9.7)	57.3 (9.5)	
Median [Q1, Q3]	59.5 [51,65]	58.5 [48,64]	59 [51,65]	
Range	36, 75	38, 76	36, 76	
Any education outside US? n (%)				.57^a^
Yes	19 (31.7)	23 (38.3)	42 (35)	
No	41 (68.3)	37 (61.7)	78 (65)	
Total	60	60	120	
If yes: highest level of education outside US n (%)				.9^a^
<12 y	10 (52.6)	12 (52.2)	22 (52.4)	
HS/GED	4 (21.1)	6 (26.1)	10 (23.8)	
2‐y technical degree	2 (10.5)	3 (13)	5 (11.9)	
4‐y Bachelor's degree	1 (5.3)	2 (8.7)	3 (7.1)	
Masters/doctorate degree	1 (5.3)	0 (0)	1 (2.4)	
Refused	1 (5.3)	0 (0)	1 (2.4)	
Total	19	23	42	
Primary language n (%)				.95^a^
English	16 (26.7)	15 (25)	31 (25.8)	
Spanish	23 (38.3)	22 (36.7)	45 (37.5)	
English and Spanish	21 (35)	23 (38.3)	44 (36.7)	
Total	60	60	120	
Which best describes your Hispanic/Latino heritage? n (%)				.96^a^
Mexican	54 (90)	52 (86.7)	106 (88.3)	
Puerto Rican	2 (3.3)	2 (3.3)	4 (3.3)	
Colombian	0 (0)	1 (1.7)	1 (0.8)	
Central America	0 (0)	1 (1.7)	1 (0.8)	
More than one heritage	2 (3.3)	2 (3.3)	4 (3.3)	
Don't know	2 (3.3)	1 (1.7)	3 (2.5)	
Refused	0 (0)	1 (1.7)	1 (0.8)	
Total	60	60	120	
Were you born in the United States? n (%)				.71^a^
Yes	36 (60)	33 (55.9)	69 (58)	
No	24 (40)	26 (44.1)	50 (42)	
Total	60	59	119	
Generation that best applies to you n (%)				.85^a^
1st generation	23 (38.3)	26 (43.3)	49 (40.8)	
2nd generation	9 (15)	7 (11.7)	16 (13.3)	
3rd generation	10 (16.7)	7 (11.7)	17 (14.2)	
4th generation	7 (11.7)	4 (6.7)	11 (9.2)	
5th generation	9 (15)	13 (21.7)	22 (18.3)	
Don't know	1 (1.7)	2 (3.3)	3 (2.5)	
N/A	1 (1.7)	1 (1.7)	2 (1.7)	
Total	60	60	120	
Insurance n (%)				.17^a^, ^c^
Through employer	2 (3.3)	9 (15)	11 (9.2)	
Medicaid	27 (45)	22 (36.7)	49 (40.8)	
Medicare	13 (21.7)	11 (18.3)	24 (20)	
Other	18 (30.0)	19 (31.2)	37 (30.8)	
Total	60	60	120	

^a^ *P* value Fisher's exact test to compare usual (PN) to enhanced (PN+) patient navigation.

^b^ *P* value Mann‐Whitney test to compare usual (PN) to enhanced (PN+) patient navigation.

^c^ Multiple selections allowed for this variable.

### 3.1 Participant encounters with *promotoras*

There were no significant differences in the average number of *promotora‐*accompanied clinic or hospital appointments between PN+ participants (mean 2.4 visits; SD 0.9; minimum [min] accompanied visits 1, maximum [max] 6) and PN participants (2.1 SD 0.7; min 1, max 4). For telephone calls with participants, *promotoras* made or received 1,274 calls for PN+ (n = 60, ie, all PN+; min 1 call, max 42 calls) compared to 107 calls for PN (n = 36 of 60; min 1, max 6).

Table 3 shows QoL at baseline and 6‐month follow‐up for the Intervention (PN+) vs Control (PN) groups. There were no significant differences between groups for FACT‐G, FACT‐B, or their individual subscales at baseline. PN participants' scores on all scales decreased between baseline and 6 months, while PN+ participants' scores improved. All scores were significantly different at 6 months (*P* = .007‐.04), except for the FACT‐G physical well‐being (PWB) subscale (*P* = .11).

**TABLE 3:** Quality of life (QoL) at baseline and 6‐mo follow‐up by study group

Scales/domains	Baseline		6‐mo follow‐up	
	Mean (SD)	*P*‐value^a^	Mean (SD)	*P*‐value^a^
General cancer QoL (FACT‐G)				
PN	84.7 (14)	0.16	77.3 (16.2)	.02
PN+	79.4 (19)		85.4 (15.9)	
Breast cancer‐related QoL (FACT‐B)				
PN	110.7 (17.3)	0.11	100.8 (20.3)	.01
PN+	103.4 (24.4)		112.1 (20)	
Physical well‐being				
PN	24.1 (3.7)	0.06	22 (5.6)	.11
PN+	22.1 (5.5)		23.6 (4.9)	
Social/family well‐being				
PN	19.8 (5.5)	0.9	18.6 (5.5)	.04
PN+	19.6 (6.8)		20.6 (5.7)	
Emotional well‐being				
PN	20.2 (3.9)	0.11	18.4 (4.7)	.03
PN+	18.9 (4.8)		20.3 (3.7)	
Functional well‐being				
PN	20.6 (5)	0.16	18.4 (5.1)	.01
PN+	18.9 (6.3)		21 (5.5)	
Breast cancer‐specific subscale				
Usual	26 (5.1)	0.23	23.4 (6.2)	.007
PN+	24.1 (6.9)		26.7 (5.8)	

Abbreviations: FACT‐B, Functional Assessments of Cancer Therapy‐Breast; FACT‐G, Functional Assessments of Cancer Therapy‐General.

^a^ Mann‐Whitney test to compare usual (PN) to enhanced (PN+) patient navigation.

Table 3 also shows the estimated intervention effect on each outcome at 6 months after sensitivity analysis. Results were similar to baseline/6‐month comparisons described above, with significant effects for both FACT‐G and FACT‐B [Coefficients (coeff)/SE 7.9/3.1; *P* = .012 and 10.9/3.9; *P* = .006, respectively]. Almost all subscales likewise showed significant intervention effects (*P* = .006‐.042). Again, there was no difference between groups for the PWB subscale (coeff/SE 1.5/1.0; *P* = .16); there was a trend toward significance in the SWB subscale (2.1/1.1; *P* = .06). None of the demographic and intervention covariates added to the model affected the direction, magnitude, or statistical significance of the Coeff for the intervention.

When comparing the intervention effect on FACT‐G specifically between PN+ participants and PN participants who chose *not* to contact a *promotora*, we found no significant effect (data not shown).

## 4 DISCUSSION

This study hoped to shift the paradigm toward the ideal that, not only do cancer patients need to be cured of cancer, they also can achieve improved QoL in survivorship through Patient Navigation. We evaluated the impact of enhanced patient navigation (Intervention; PN+) compared to Control (PN) on QoL in Latina BCS and found a significant effect after 6 months: improved general cancer (FACT‐G) and breast cancer‐specific (FACT‐B) QoL scores in the PN+ group, as well as a significant difference from the PN group. When broken down by subscale score, we found significant differences for almost all QoL subscales at 6 months as measured by FACT‐G. These differences were likely due to the *promotoras*’ efforts; they played a key role in walking PN+ participants through any barriers they encountered. Not only did *promotoras* assist with clinical needs, they also provided social and emotional support. Their expertise helped PN+ participants address issues like transportation, housing, education, and access to insured health care, and facilitated introduction to community support systems for nutrition and fitness.

The exception to the differences seen between PN+ and PN participants was the PWB subscale score. This was likely due to changes in participant PWB over the study period, in opposite directions: the PN+ group showed a small PWB score improvement from mean (SD) of 22.1 (5.5) at baseline to 23.6 (4.9) at 6 months. In contrast, the PN group showed a larger worsening of PWB score—24.1 (3.7) and 22 (5.6) at baseline and 6 months, respectively. Since the PN group had a higher (albeit nonsignificantly different) PWB score at baseline, these changes effectively canceled out any differences between groups at 6 months. Long‐term follow‐up in a comparable sample at 12 months or more would be beneficial to determine if any PWB differences present themselves.

In addition, the estimated intervention effect was also not significant for the SWB at 6 months [coeff (SE) 2.1 (1.1); *P* = .06]. Several factors beyond the scope of this study could contribute to these scores, including intervention dose: for example, length and number of phone calls with *promotoras* between PN+ participant subgroups may differ by any number of covariates (eg, age, stage at diagnosis, baseline QoL scores, etc). The lack of difference between groups when comparing PN+ participants who had telephone contact with *promotoras* and PN participants who did not avail of this service also needs to be explored. Other factors include participant fitness, stress levels, and support systems at the two time points. These will be addressed in future studies.

Except for PWB, *promotora* assistance positively and significantly influenced QoL in our sample. These robust results are an important addition to the literature showing that Patient Navigation has positive effects on QoL in Latina BCS, which has not been definitively shown in previous studies.^36^, ^55^

### 4.1 Limitations and strengths

The Staying Healthy intervention has several unique features not present in other trials. For example, we designed the intervention using an iterative user‐centered design process developed in a previous study,^49^ which involved cancer survivors experiencing numerous comorbid conditions. We included an extensive assessment battery that allowed us to examine a wide array of issues related to the value of Patient Navigation in Latina BCS. We conducted the trial of our intervention to adhere, as much as possible, to the CONSORT Statement for social and psychological randomized clinical trials.^51^ Limitations include exclusion of BCS with stage 4 or recurrent disease and BCS undergoing treatment at the time of the study. Also excluded were normal‐weight patients who may also suffer from comorbidities (eg, high cholesterol and hypertension), impacting their QoL despite their lower BMI.^56^ The program was also specifically targeted to Latina BCS. However, future studies may adapt it to other languages and cultures and expand Patient Navigation services to earlier time points in the BCS experience.

### 4.2 Future work

Future reports will focus on comparing PN+ to PN on rates of treatment adherence, cancer and comorbidity screening compliance, and QoL interactions with covariates like supportive care needs, perceived efficacy in patient‐physician interactions, health behavior change, level of distress, worry interference, self‐efficacy, satisfaction with health care, sociodemographic factors, life events/stressors, medical comorbidities, and health‐care utilization. It will also be important to understand what role behavioral/lifestyle change referrals (ie, nutrition and exercise) play in impacting Latina BCS well‐being. Evidence has shown that survivors who make lifestyle changes after their diagnosis feel better, are less fatigued, and reduce their risk of a cancer recurrence.^57^, ^58^ The finding of improved QoL among our PN+ group participants at 6 months supports this. In addition, survivors and their HCPs understand the challenges posed by a cancer diagnosis and the importance of ongoing adherence to HCP recommendations to potentially increase survival likelihood and/or reduce recurrent or new cancer risk.^59^ Additional studies to promote synergistic communication and action by HCPs and survivors are needed.^14^, ^60^, ^61^, ^62^

The Staying Healthy program has the potential to become a national model of survivorship care for improving QoL in Latina BCS. Future studies involving Patient Navigation earlier in the BCS experience (ie, immediately after diagnosis and through treatment), as well as BCS with metastatic and recurrent disease and those from other underserved groups will explore the program's applicability to a wider BCS population.

## CONFLICT OF INTEREST

None of the authors have any conflict of interest to declare.

## AUTHOR CONTRIBUTIONS

AGR conceptualized and supervised the conduct of the study; EM supervised data collection; EM and DLP conducted statistical analyses; DLP and AP prepared the manuscript for submission; all authors reviewed and critiqued the manuscript for content.

## ETHICAL STATEMENT

This study was approved by the Institutional Review Board at UT Health San Antonio and the Mays Cancer Center Protocol Review Committee, and conforms to the *US Federal Policy for the Protection of Human Subjects*.

## Data Availability

The data that support the findings of this study are available on request from the corresponding author. The data are not publicly available due to privacy or ethical restrictions.
